# Supplementary material for: Engineered long-acting Irisin-albumin binding domain fusion protein for enhanced anti-inflammatory efficacy in lipopolysaccharide-induced systemic inflammation
Source: Commun Biol. 2025 Nov 17;8:1592. doi: 10.1038/s42003-025-09000-z (PMC12623720; doi:10.1038/s42003-025-09000-z)
Supplement: Supplementary file 1 — Supplementary Information [file 42003_2025_9000_MOESM1_ESM.pdf]

Supplementary Information for

Engineered long-acting Irisin-albumin binding domain fusion protein for enhanced anti-inflammatory efficacy in lipopolysaccharides-induced systemic inflammation

Jicun Zhu[1,9], Yujie Zhang[2,3,9], Xinwei Wang[2,9], Lei Peng[2], Xiaojia Ma[2], Zan Qiu[2], Zirui Kang[2], Fangyuan Zheng[2], Xiaoyu Zhang[4], Mengyuan Song[4], Jia Du[5], Yuan Shi[6], Lie Yu[7*], Chenxi Gu[8*], Jianxiang Shi[2*]

[1]Department of Pharmacy, The First Affiliated Hospital of Zhengzhou University, Zhengzhou, 450052, China. [2]Henan Institute of Medical and Pharmaceutical Sciences, Zhengzhou University, Zhengzhou, 450052, China. [3]Henan Key Laboratory of Microbiome and Esophageal Cancer Prevention and Treatment & Henan Key Laboratory of Cancer Epigenetics, The First Affiliated Hospital (College of Clinical Medicine) of Henan University of Science and Technology, Luoyang, 471003, China. [4]School of Basic Medical Sciences, Zhengzhou University, Zhengzhou, 450001, China. [5]College of Public Health, Zhengzhou University, Zhengzhou, 450001, China. [6]Anyang Tumor Hospital, The Affiliated Anyang Tumor Hospital of Henan University of Science and Technology, Anyang, 455000, China. [7]Department of Neurology, The First Affiliated Hospital of Zhengzhou University, Zhengzhou, 450052, China. [8]Department of Orthopaedic Surgery, The First Affiliated Hospital of Zhengzhou University, Zhengzhou, 450052, China. [9]These authors contributed equally: Jicun Zhu, Yujie Zhang, Xinwei Wang.

Contains:

Tables S1-S3

Figure S1-S10

Table S1 Concentrations of Irisin and ABD-Irisin fusion protein in mice plasma (mean ± S.D.) (ng/mL)

Time (h)	Irisin			ABD-Irisin		
	100 μg/kg	500 μg/kg	1000 μg/kg	100 μg/kg	500 μg/kg	1000 μg/kg
0	0.75 ±0	0.75 ±0	0.75 ±0	0.75 ±0	0.75 ±0	0.75 ±0
0.5	27.05 ±7.09	176.89 ±18.31	281.49 ±21.47	233.52 ±10.0****	349.39 ±2.63****	460.87 ±23.68****
1	10.63 ±3.47	55.64 ±8.85	95.12 ±9.21	303.60 ±8.62****	435.57 ±19.25****	669.71 ±20.95****
2	6.68 ±0.96	31.51 ±0.62	53.04 ±2.58	271.01 ±22.68****	423.47 ±16.92****	594.73 ±20.09****
4	3.77 ±1.03	13.47 ±1.03	29.13 ±2.58	241.49 ±20.72****	372.62 ±19.84****	564.44 ±12.99****
8	0.97 ±0.17	4.88 ±0.46	11.68 ±0.43	220.37 ±11.93****	358.35 ±13.56****	450.78 ±30.37****
12	0.75 ±0	0.75 ±0	0.75 ±0	69.07 ±10.13****	91.08 ±12.22****	128.90 ±14.42****
24	0.75 ±0	0.75 ±0	0.75 ±0	40.45 ±1.45****	27.83 ±5.66****	31.06 ±9.54****

Note: Repeated measures ANOVA was employed to evaluate differences in the concentrations of Irisin and ABD-Irisin fusion protein in mouse plasma at the same dose (n = 4 per dose group). Data are presented as mean ± S.D. Statistical significance: *$P < 0.05$, **$P < 0.01$, ***$P < 0.001$, ****$P < 0.0001$.

Table S2 Body weights in mice during drug administration (mean ± S.D.) (g)

Time (d)	PBS	LPS	Irisin			ABD-Irisin		
			100 μg/kg	500 μg/kg	1000 μg/kg	100 μg/kg	500 μg/kg	1000 μg/kg
1	19.24 ±0.98	19.14 ±0.42	19.28 ±0.59	19.36 ±0.25	19.08 ±0.85	19.56 ±0.65	19.22 ±0.41	19.04 ±0.57
2	20.04 ±0.55	19.18 ±0.47	19.54 ±0.68	19.76 ±0.71	19.42 ±0.54	20.12 ±0.55	19.2 ±0.78	19.14 ±0.52
3	21.08 ±0.48	19.12 ±0.52**	20.04 ±1.04	20.00 ±0.73	19.86 ±1.09	20.38 ±1.14	19.54 ±0.88	19.74 ±0.53
4	21.54 ±0.27	19.22 ±0.34****	20.26 ±1.06	20.52 ±0.36	20.60 ±1.30	20.5 ±1.35	20.24 ±1.16	20.08 ±0.64
5	21.74 ±0.21	19.78 ±0.55**	20.74 ±0.31	21.38 ±0.75$^{\#}$	21.38 ±1.16$^{\#}$	21.32 ±0.49$^{\#}$	20.86 ±0.99	21.00 ±0.46
6	22.1 ±0.29	19.86 ±0.34****	20.84 ±0.67	21.54 ±0.21$^{\#}$	21.60 ±1.27$^{\#\#}$	21.52 ±0.57$^{\#}$	20.88 ±1.1	21.12 ±0.62

Note: Repeated measures ANOVA was employed to evaluate body weight differences among experimental groups of mice during the administration period (n = 5 per group). *: LPS *vs* PBS (*$P < 0.05$, **$P < 0.01$, ***$P < 0.001$, ****$P < 0.0001$, ns: not significant), #: Irisin/ABD-Irisin *vs* LPS ($^{\#}P < 0.05$, $^{\#\#}P < 0.01$, $^{\#\#\#}P < 0.001$, $^{\#\#\#\#}P < 0.0001$, ns: not significant).

Table S3 List of antibodies

Antibody	Company/Origin	Catalog number
Anti-FNDC5/irisin	Abcam, Waltham, MA, USA	ab174833
Recombinant Anti-His Tag Mouse mAb	ServiceBio Technology Co., Ltd. Wuhan, China	GB151251
Anti-Albumin Rabbit pAb	ServiceBio Technology Co., Ltd. Wuhan, China	GB11319
Anti-LBP Rabbit pAb	ServiceBio Technology Co., Ltd. Wuhan, China	GB113205
Anti-CD14 Mouse mAb	ServiceBio Technology Co., Ltd. Wuhan, China	GB14023
Anti-TLR4 Mouse mAb	ServiceBio Technology Co., Ltd. Wuhan, China	GB12186
Anti-MyD88 Mouse mAb	ServiceBio Technology Co., Ltd. Wuhan, China	GB12269
Anti-NF-κB p65 Mouse mAb	ServiceBio Technology Co., Ltd. Wuhan, China	GB12997
Anti-IL-1 beta Mouse mAb	ServiceBio Technology Co., Ltd. Wuhan, China	GB122059
Anti-IL-10 Mouse mAb	ServiceBio Technology Co., Ltd. Wuhan, China	GB12108
Goat Anti-Mouse Ig G	HuaBio Co., Ltd., Hangzhou, China	G1006-1
Goat Anti-Rabbit Ig G	HuaBio Co., Ltd., Hangzhou, China	HA1012

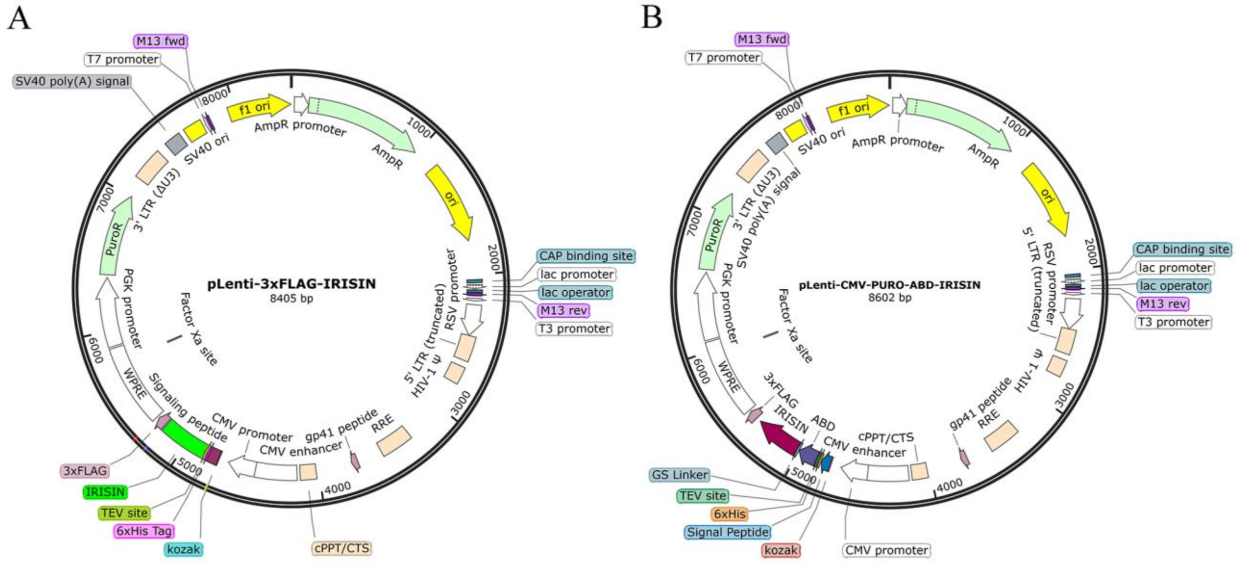

Figure S1 Schematic diagram of P3F-Irisin and P3F-ABD-Irisin plasmid constructs.

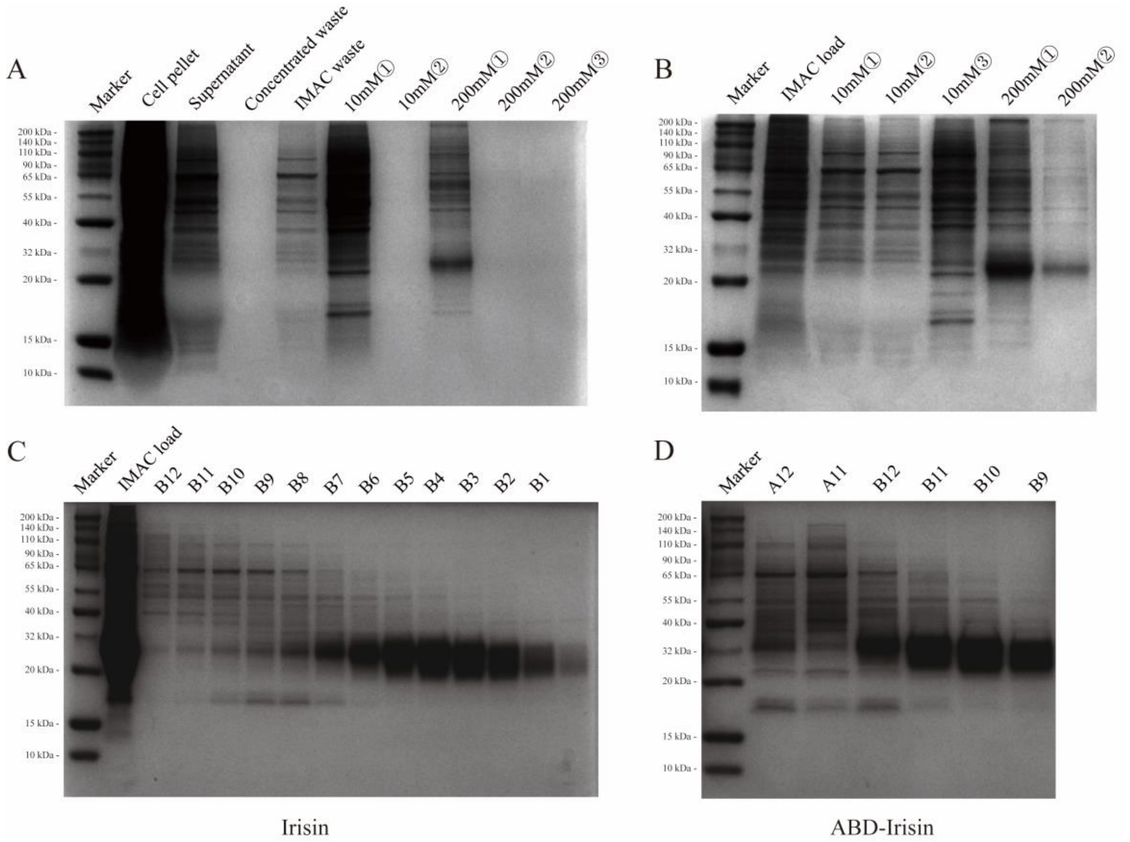

Figure S2 Expression and purification of Irisin and ABD-Irisin fusion proteins. A Affinity chromatography of Irisin protein. **B** Affinity chromatography of ABD-Irisin fusion protein. **C** Gel filtration chromatography of Irisin protein. **D** Gel filtration chromatography of ABD-Irisin fusion protein.

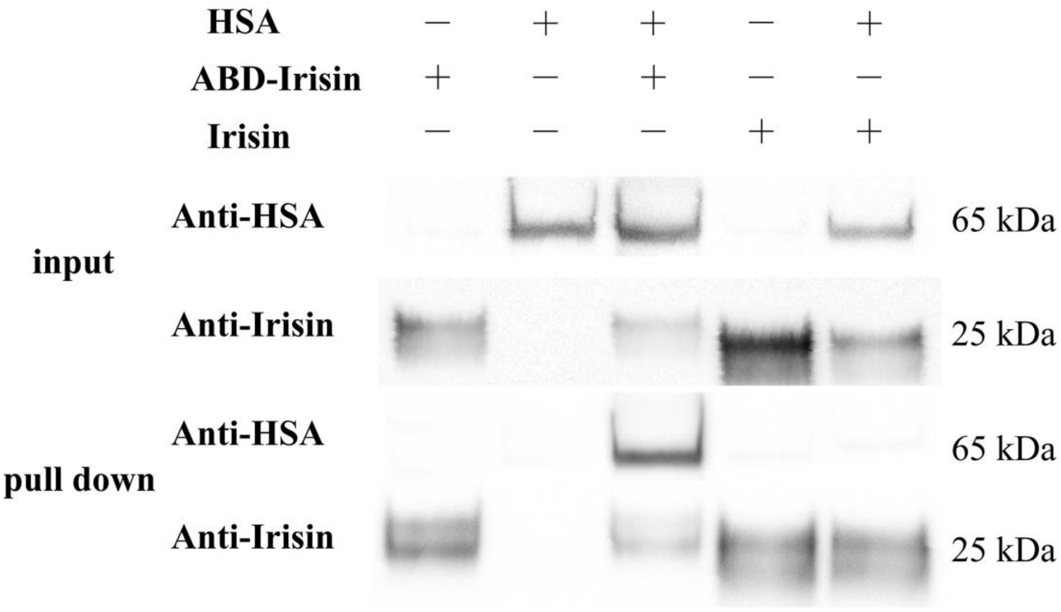

Figure S3 Western blot analysis of pull-down and input proteins. Input lanes: Whole-cell lysates containing 65 kDa HSA were probed with anti-HAS and anti-Irisin. All lanes show the expected bands, confirming the presence of both HSA and the Irisin moiety in the respective samples. Pull-down lanes: Ni^{2+} -magnetic beads loaded with His-tagged Irisin or ABD-Irisin were incubated with HSA. After stringent washing, only the ABD-Irisin sample retained HSA. Anti-Irisin blot verifies comparable loading of the His-tagged bait proteins. The co-retention of HSA specifically with ABD-Irisin demonstrates that the ABD domain within ABD-Irisin folds correctly and binds HSA in vitro.

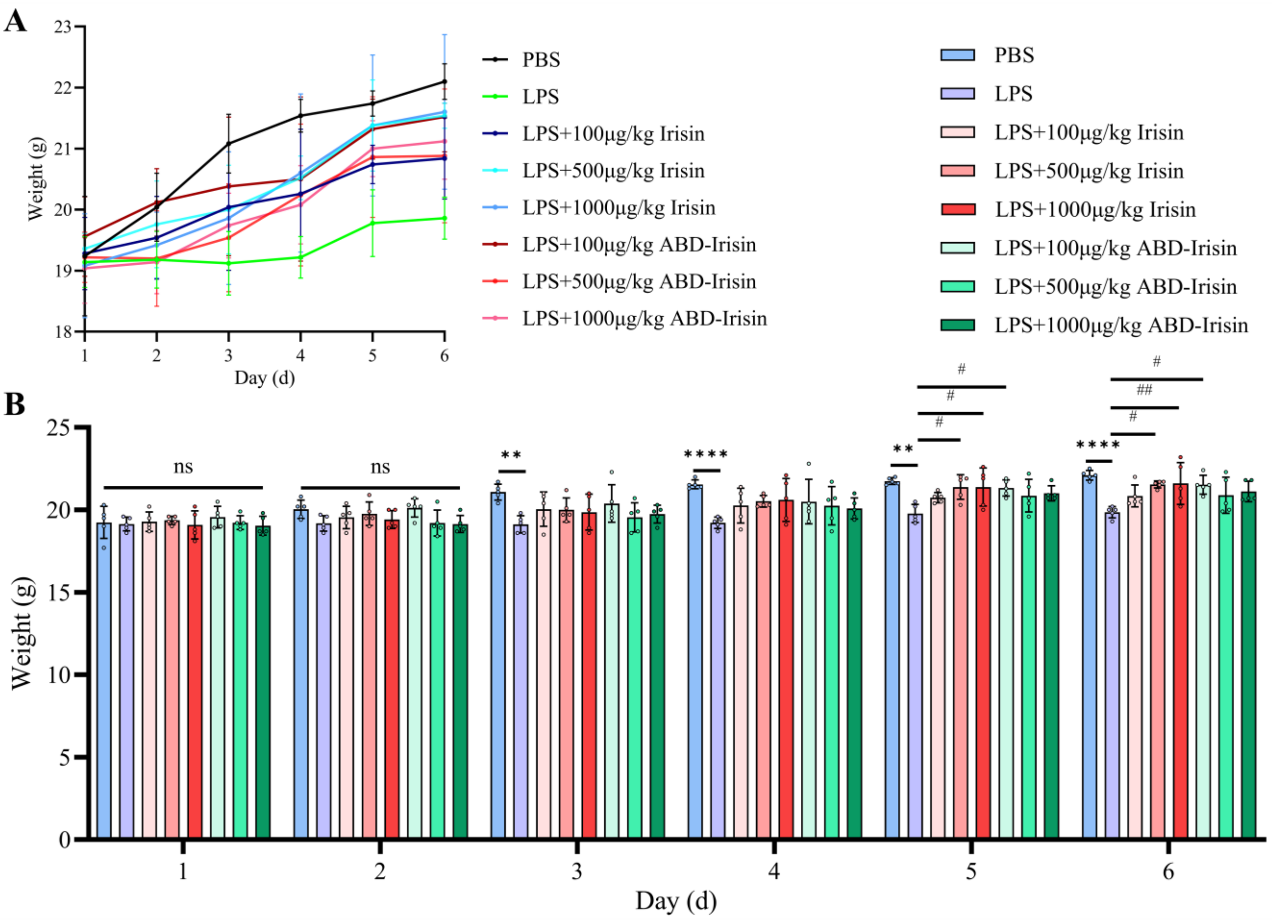

Figure S4 Comparison of Body weight in mice among different groups (n = 5 per group). *: LPS *vs* PBS (*$P < 0.05$, **$P < 0.01$, ***$P < 0.001$, ****$P < 0.0001$, ns: not significant), #: Irisin/ABD-Irisin *vs* LPS (#$P < 0.05$, ##$P < 0.01$, ###$P < 0.001$, ####$P < 0.0001$, ns: not significant).

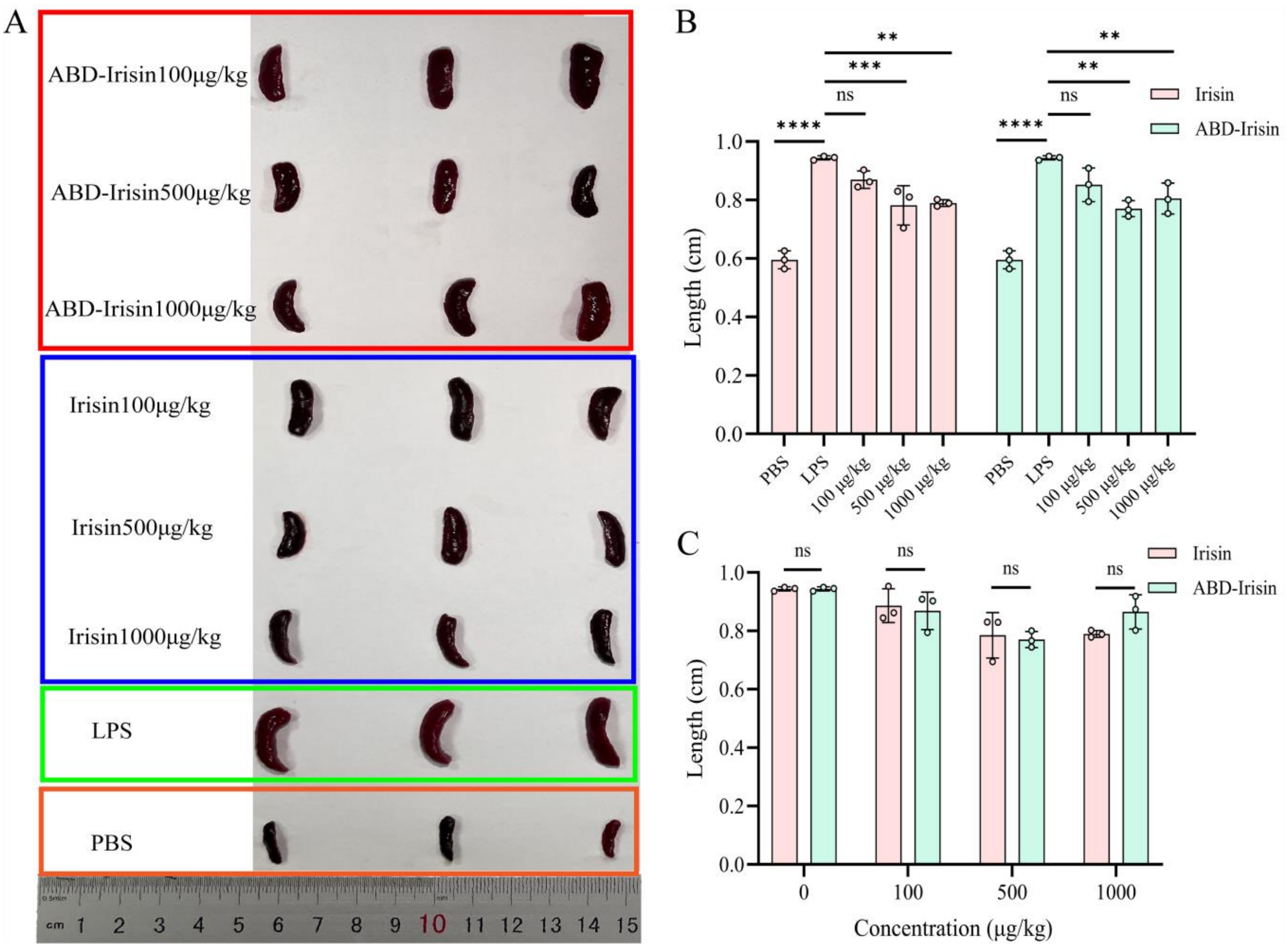

Figure S5 Comparison of spleen length in mice among different groups. A Representative gross anatomy of spleens in different treatment groups. **B** Quantitative analysis of spleen length across treatment groups. **C** Spleen length comparison among protein intervention groups at the same concentration (n = 3 per group). Data are presented as mean ± S.D. Statistical significance: **P* < 0.05, ***P* < 0.01, ****P* < 0.001, *****P* < 0.0001, ns: not significant.

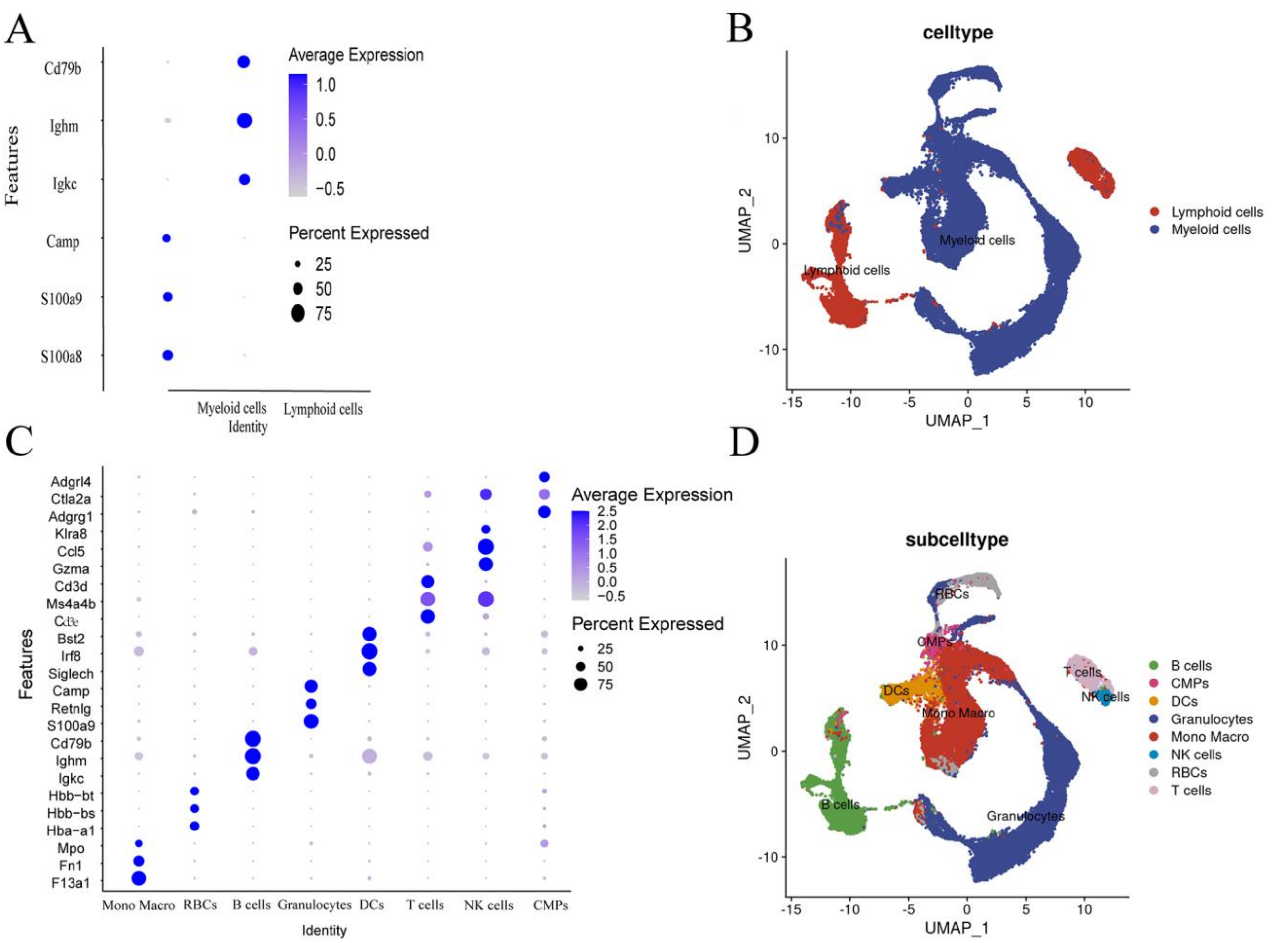

Figure S6 Single-cell RNA-seq revealed distinct cell clusters. A Top 3 marker genes of major cell clusters. **B** Clustering analysis of major cell clusters. **C** Top 3 marker genes of cell sub-clusters. **D** Clustering analysis of cell sub-clusters.

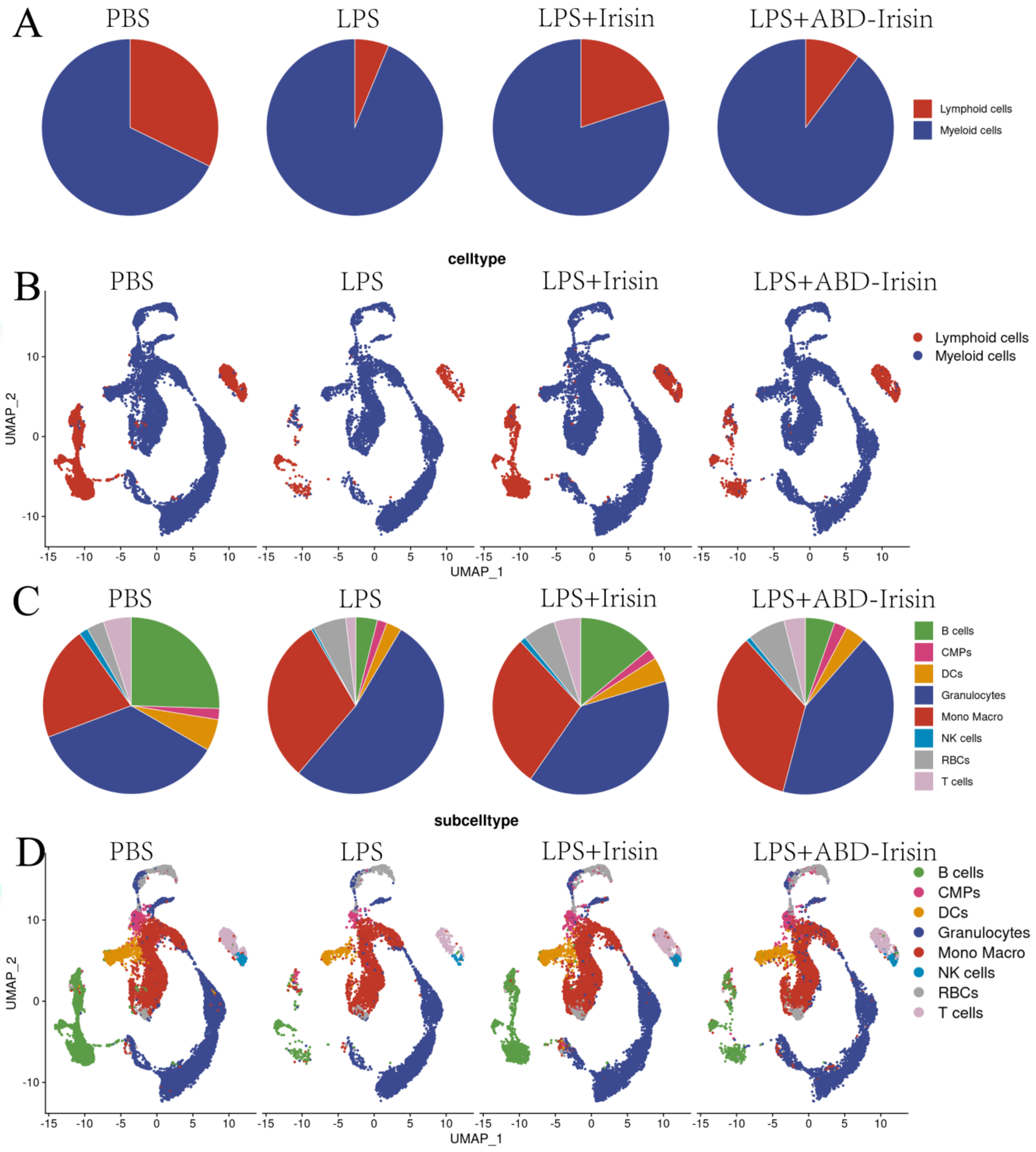

Figure S7 Distributional differences of bone marrow leukocytes in mice across treatment groups. A Major cell population distribution. **B** Clustering of major cell populations. **C** Subset distribution. **D** Subset clustering patterns.

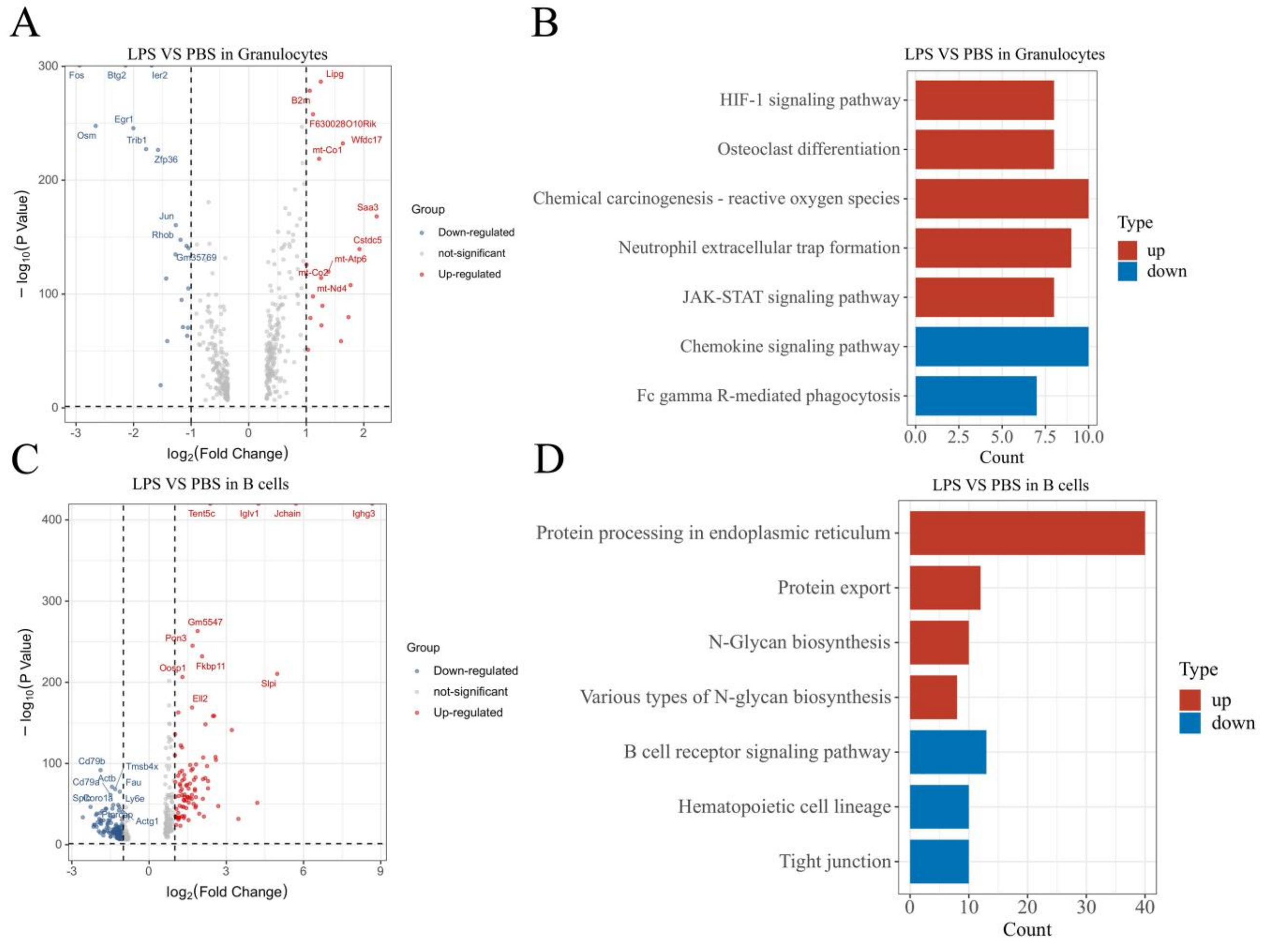

Figure S8 Differential gene expression and KEGG signaling pathways in granulocytes and B cells: LPS *vs* PBS.

A DEGs in granulocytes. **B** Enriched KEGG pathways in granulocytes. **C** DEGs in B cells. **D** Enriched KEGG pathways in B cells.

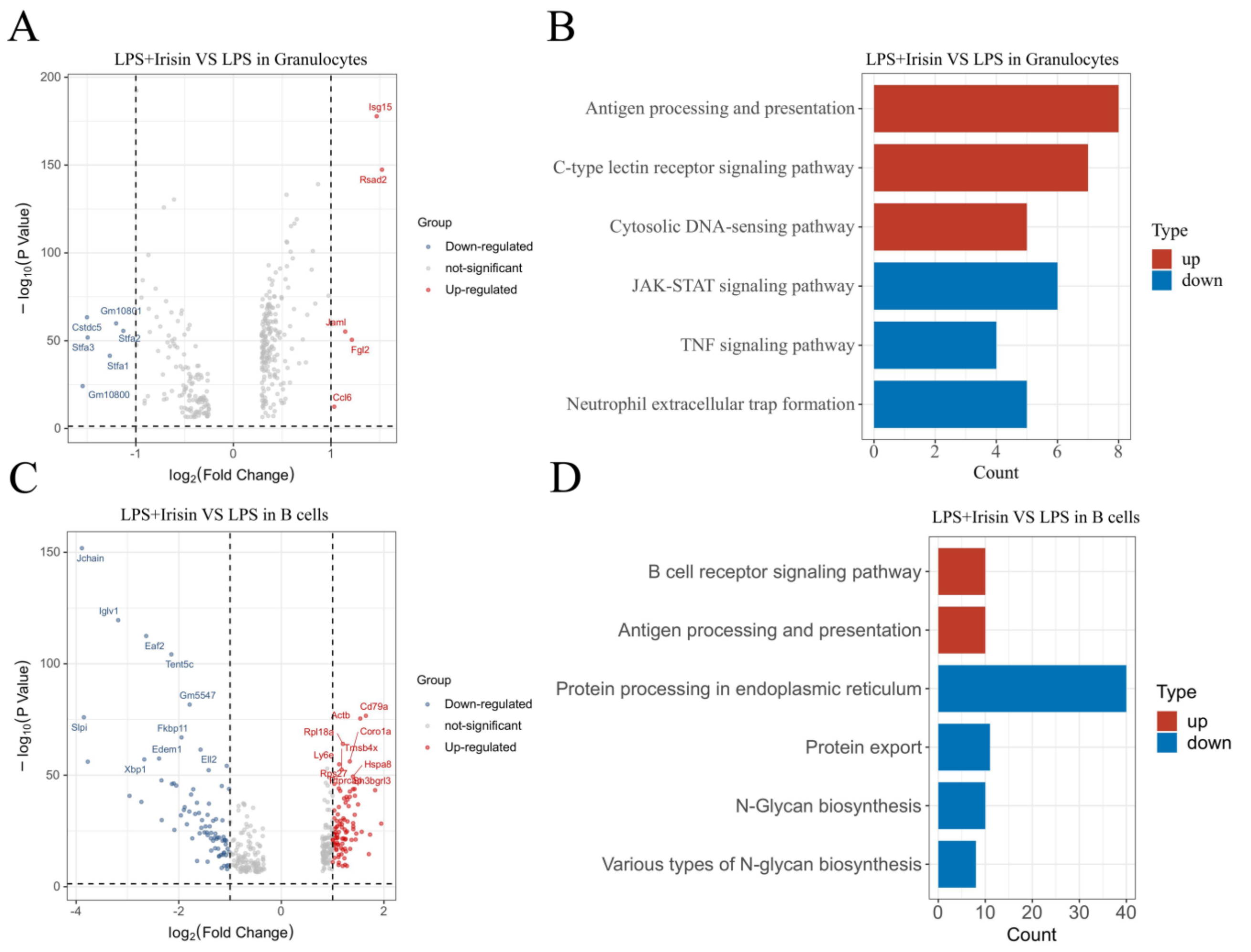

Figure S9 Differential gene expression and KEGG signaling pathways in granulocytes and B cells: LPS+Irisin *vs* LPS. A DEGs in granulocytes. **B** Enriched KEGG pathways in granulocytes. **C** DEGs in B cells. **D** Enriched KEGG pathways in B cells.

Figure S10

Fig. 1A

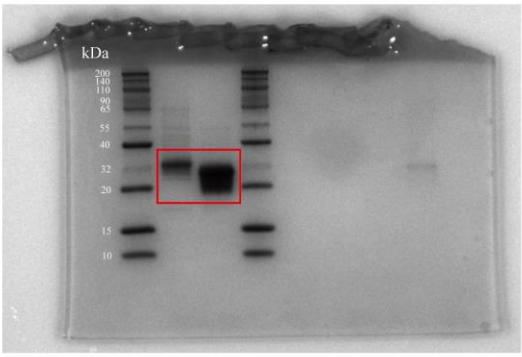

Fig. 1B

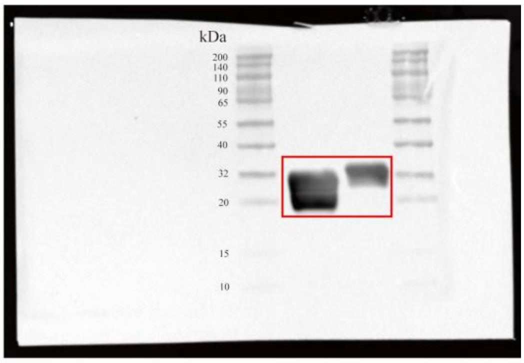

Fig. 1C

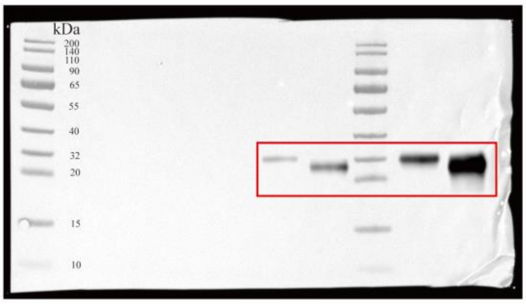

Supplementary Fig. S2A

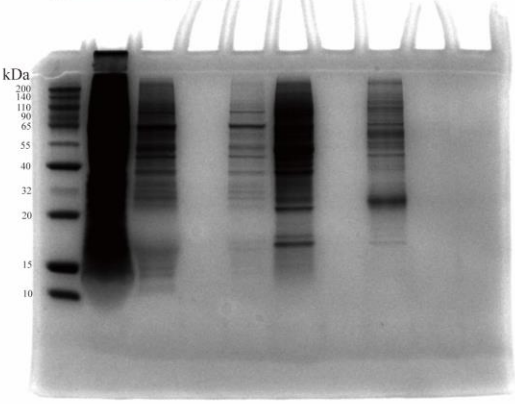

Supplementary Fig. S2B

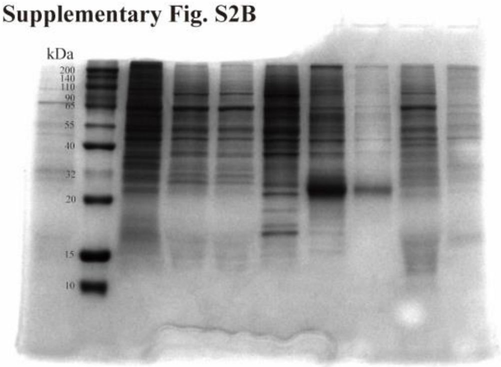

Supplementary Fig. S2C

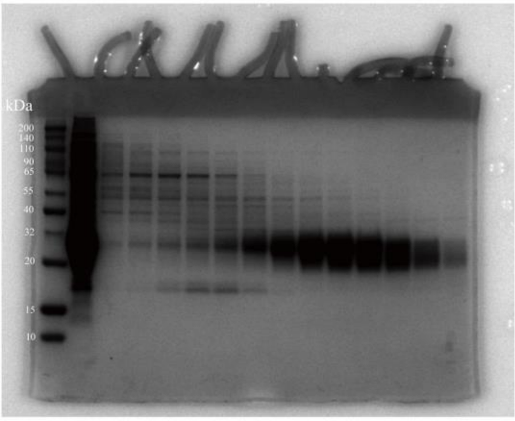

Supplementary Fig. S2D

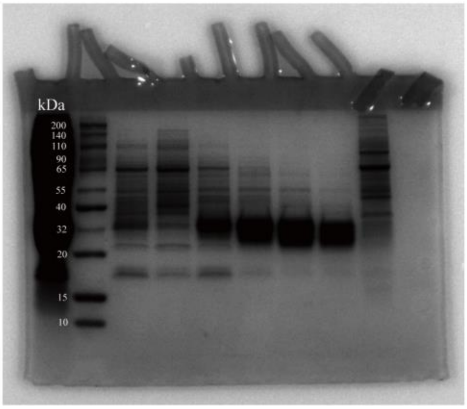

Figure S10 Uncropped/unedited images of blots.
