# Supplementary material for: Engineered long-acting Irisin-albumin binding domain fusion protein for enhanced anti-inflammatory efficacy in lipopolysaccharide-induced systemic inflammation
Source: Commun Biol. 2025 Nov 17;8:1592. doi: 10.1038/s42003-025-09000-z (PMC12623720; doi:10.1038/s42003-025-09000-z)
Supplement: Supplementary file 4 — Reporting Summary [file 42003_2025_9000_MOESM4_ESM.pdf]

nature portfolio

Corresponding author(s): DARP: COMMSBIO-25-3788A

Last updated by author(s): Sep 11, 2025

# Reporting Summary

Nature Portfolio wishes to improve the reproducibility of the work that we publish. This form provides structure for consistency and transparency in reporting. For further information on Nature Portfolio policies, see our Editorial Policies and the Editorial Policy Checklist.

## Statistics

For all statistical analyses, confirm that the following items are present in the figure legend, table legend, main text, or Methods section.

| n/a | Confirmed | |
|---|---|---|
| ☐ | ☒ | The exact sample size (*n*) for each experimental group/condition, given as a discrete number and unit of measurement |
| ☐ | ☒ | A statement on whether measurements were taken from distinct samples or whether the same sample was measured repeatedly |
| ☐ | ☒ | The statistical test(s) used AND whether they are one- or two-sided<br>*Only common tests should be described solely by name; describe more complex techniques in the Methods section.* |
| ☒ | ☐ | A description of all covariates tested |
| ☐ | ☒ | A description of any assumptions or corrections, such as tests of normality and adjustment for multiple comparisons |
| ☐ | ☒ | A full description of the statistical parameters including central tendency (e.g. means) or other basic estimates (e.g. regression coefficient) AND variation (e.g. standard deviation) or associated estimates of uncertainty (e.g. confidence intervals) |
| ☐ | ☒ | For null hypothesis testing, the test statistic (e.g. *F*, *t*, *r*) with confidence intervals, effect sizes, degrees of freedom and *P* value noted<br>*Give P values as exact values whenever suitable.* |
| ☒ | ☐ | For Bayesian analysis, information on the choice of priors and Markov chain Monte Carlo settings |
| ☒ | ☐ | For hierarchical and complex designs, identification of the appropriate level for tests and full reporting of outcomes |
| ☒ | ☐ | Estimates of effect sizes (e.g. Cohen's *d*, Pearson's *r*), indicating how they were calculated |

*Our web collection on statistics for biologists contains articles on many of the points above.*

## Software and code

Policy information about availability of computer code

| | |
|---|---|
| Data collection | N/A |
| Data analysis | N/A |

For manuscripts utilizing custom algorithms or software that are central to the research but not yet described in published literature, software must be made available to editors and reviewers. We strongly encourage code deposition in a community repository (e.g. GitHub). See the Nature Portfolio guidelines for submitting code & software for further information.

## Data

Policy information about availability of data

All manuscripts must include a data availability statement. This statement should provide the following information, where applicable:

- Accession codes, unique identifiers, or web links for publicly available datasets
- A description of any restrictions on data availability
- For clinical datasets or third party data, please ensure that the statement adheres to our policy

All data associated with this study are provided within the paper or the Supplemental Information. The source data behind the graphs in the paper can be found in Supplementary Data 1. The fastq files of the scRNA-seq data are available from the SRA database under Project ID: PRJNA1292814. The newly generated plasmids P3F-IRISIN and P3F-ABD-IRISIN have been deposited in the WeKwikGene (Westlake Laboratory Plasmid Repository) under Barcode numbers 0002003 and 0002004, respectively. Any other data is available from the corresponding author upon request.

## Research involving human participants, their data, or biological material

Policy information about studies with human participants or human data. See also policy information about sex, gender (identity/presentation), and sexual orientation and race, ethnicity and racism.

| | |
|---|---|
| Reporting on sex and gender | N/A |
| Reporting on race, ethnicity, or other socially relevant groupings | N/A |
| Population characteristics | N/A |
| Recruitment | N/A |
| Ethics oversight | N/A |

Note that full information on the approval of the study protocol must also be provided in the manuscript.

# Field-specific reporting

Please select the one below that is the best fit for your research. If you are not sure, read the appropriate sections before making your selection.

☒ Life sciences ☐ Behavioural & social sciences ☐ Ecological, evolutionary & environmental sciences

For a reference copy of the document with all sections, see nature.com/documents/nr-reporting-summary-flat.pdf

# Life sciences study design

All studies must disclose on these points even when the disclosure is negative.

| | |
|---|---|
| Sample size | In strict adherence to the 3Rs principles (particularly Reduction, Refinement), the sample size was determined through a priori power analysis based on effect sizes from Reference [28]. |
| Data exclusions | For scRNA-seq analysis, one sample from the LPS-treated group was excluded due to poor data quality. |
| Replication | All replication experiments were successful, verifying the reproducibility of the experimental results. |
| Randomization | Mice were randomly assigned to different treatment groups using a random number generator. |
| Blinding | Blinding was not implemented during the data collection and/or analysis in this study. This is because it is a transparent observational study, and there is no risk of bias arising from knowledge of the group assignments. |

# Reporting for specific materials, systems and methods

We require information from authors about some types of materials, experimental systems and methods used in many studies. Here, indicate whether each material, system or method listed is relevant to your study. If you are not sure if a list item applies to your research, read the appropriate section before selecting a response.

### Materials & experimental systems

| n/a | Involved in the study | |
|---|---|---|
| ☐ | ☒ | Antibodies |
| ☐ | ☒ | Eukaryotic cell lines |
| ☒ | ☐ | Palaeontology and archaeology |
| ☐ | ☒ | Animals and other organisms |
| ☒ | ☐ | Clinical data |
| ☒ | ☐ | Dual use research of concern |
| ☒ | ☐ | Plants |

### Methods

| n/a | Involved in the study | |
|---|---|---|
| ☒ | ☐ | ChIP-seq |
| ☒ | ☐ | Flow cytometry |
| ☒ | ☐ | MRI-based neuroimaging |

## Antibodies

| | |
|---|---|
| Antibodies used | Anti-FNDC5/irisin (Abcam, Catalog #: ab174833), Recombinant Anti-His Tag Mouse mAb (ServiceBio, Catalog #: GB151251), Anti-Albumin Rabbit pAb (ServiceBio, Catalog #: GB11319), Anti-LBP Rabbit pAb (ServiceBio, Catalog #: GB113205), Anti-CD14 Mouse mAb (ServiceBio, Catalog #: GB14023), Anti-TLR4 Mouse mAb (ServiceBio, Catalog #: GB12186), Anti-MyD88 Mouse mAb (ServiceBio, |

| | |
|---|---|
| | Catalog #: GB12269), Anti-NF-κB p65 Mouse mAb (ServiceBio, Catalog #: GB12997), Anti-IL-1 beta Mouse mAb (ServiceBio, Catalog #: GB122059), Anti-IL-10 Mouse mAb (ServiceBio, Catalog #: GB12108), Goat Anti-Mouse Ig G (HuaBio, Catalog #: G1006-1), Goat Anti-Rabbit Ig G (HuaBio, Catalog #: HA1012). |
| Validation | Anti-FNDC5/irisin, Species: Rabbit, Application: WB. Produced recombinantly (animal-free) for high batch-to-batch consistency and long term security of supply.<br>Recombinant Anti-His Tag Mouse mAb, Species: Mouse, Application: Tag antibody can be used in a variety of applications, including ChIP, CoIP, ELISA, FC, IF, IHC, IP, WB and so on.<br>Anti-Albumin Rabbit pAb, Species: Rabbit, Application: WB. Serum albumin, the main protein of plasma, has a good binding capacity for water, $Ca^{2+}$, $Na^+$, $K^+$, fatty acids, hormones, bilirubin and drugs. Its main function is the regulation of the colloidal osmotic pressure of blood.<br>Anti-LBP Rabbit pAb, Species: Rabbit, Application: WB. LBP is an acute-phase protein, predominantly synthesized by hepatocytes. LBP binds to the lipid A moiety of bacterial lipopolysaccharides (LPS), a glycolipid present in the outer membrane of all Gram-negative bacteria, and acts as an affinity enhancer for CD14, facilitating its association with LPS.<br>Anti-CD14 Mouse mAb, Species: Rabbit, Application: IHC/IF. CD14 serves as a multifunctional lipopolysaccharide receptor, and is released to the serum both as a secreted and enzymatically cleaved GPI-anchored form. CD14 binds lipopolysaccharide molecule in a reaction catalyzed by LBP, an acute phase serum protein.<br>Anti-TLR4 Mouse mAb, Species: Mouse, Application: IHC/IF. TLR4 activation leads to an intracellular signaling pathway NF-κB and inflammatory cytokine production which is responsible for activating the innate immune system. It is most well-known for LPS, a component present in many Gram-negative bacteria (e.g. Neisseria spp.) and select Gram-positive bacteria.<br>Anti-MyD88 Mouse mAb, Species: Mouse, Application: WB. MYD88 is the key adaptor protein for the Toll/IL-1R and is involved in the inflammatory response induced by IL-1, IL-18 and LPS, which are essential for innate immunity and pathogen-associated molecular pattern recognition.<br>Anti-NF-κB p65 Mouse mAb, Species: Mouse, Application: WB, HC/IF. NF-kappa-B is a pleiotropic transcription factor present in almost all cell types and is the endpoint of a series of signal transduction events that are initiated by a vast array of stimuli related to many biological processes such as inflammation, immunity, differentiation, cell growth, tumorigenesis and apoptosis.<br>Anti-IL-1 beta Mouse mAb, Species: Mouse, Application: WB. Potent pro-inflammatory cytokine Initially discovered as the major endogenous pyrogen, induces prostaglandin synthesis, neutrophil influx and activation, T-cell activation and cytokine production, B-cell activation and antibody production, and fibroblast proliferation and collagen production Promotes Th17 differentiation of T-cells.<br>Anti-IL-10 Mouse mAb, Species: Mouse, Application: HC/IF. IL-10 downregulates the expression of Th1 cytokines, MHC class II antigens, and co-stimulatory molecules on macrophages. IL-10 can block NF-κB activity, and is involved in the regulation of the JAK-STAT signaling pathway.<br>Goat Anti-Mouse Ig G, Species: Goat, Application: WB, ELISA.<br>Goat Anti-Rabbit Ig G, Species: Goat, Application: WB, ELISA. |

## Eukaryotic cell lines

Policy information about cell lines and Sex and Gender in Research

| | |
|---|---|
| Cell line source(s) | HEK-293T and HEK-293F cell lines were obtained from the Precision Medicine Laboratory cell bank at Zhengzhou University. The 3T3-L1 cell line was purchased from Cyagen Biotechnology Co., Ltd. (Guangzhou, China). |
| Authentication | HEK-293T, HEK-293F and 3T3-L1 were authenticated by short tandem repeat (STR) analysis. The detection results were compared with the standard profiles provided by ATCC to confirm the identity of the cell lines. |
| Mycoplasma contamination | All the cell lines used in this study were tested for mycoplasma contamination, and the results were all negative. |
| Commonly misidentified lines<br>(See ICLAC register) | N/A |

## Animals and other research organisms

Policy information about studies involving animals; ARRIVE guidelines recommended for reporting animal research, and Sex and Gender in Research

| | |
|---|---|
| Laboratory animals | Male C57BL/6J mice (n = 65, 6 weeks old) were purchased from Zhejiang Vital River Laboratory Animal Technology Co., Ltd. (Pinghu, China) and maintained under specific pathogen-free (SPF) conditions at the Experimental Animal Center of Zhengzhou University. |
| Wild animals | The study did not involve wild animals. |
| Reporting on sex | No sex-based analysis was performed in this study, as the research focus was on the anti-inflammatory effect of the ABD-Irisin fusion protein, and sex differences were considered to have a minimal impact on the results. |
| Field-collected samples | The study did not involve samples collected from the field. |
| Ethics oversight | All animal experiments were approved by the Ethics Committee of Zhengzhou University Laboratory Animal Center (approval number: ZZU-LAC20230825[03]). |

Note that full information on the approval of the study protocol must also be provided in the manuscript.

## Plants

| | |
|---|---|
| Seed stocks | N/A |
| Novel plant genotypes | N/A |
| Authentication | N/A |
